# Machine learning-based radiomics model to predict benign and malignant PI-RADS v2.1 category 3 lesions: a retrospective multi-center study

**DOI:** 10.1186/s12880-023-01002-9

**Published:** 2023-03-29

**Authors:** Pengfei Jin, Junkang Shen, Liqin Yang, Ji Zhang, Ao Shen, Jie Bao, Ximing Wang

**Affiliations:** 1 Department of Radiology, The Cancer Hospital of the University of Chinese Academy of Science (Zhejiang Cancer Hospital), Institute of Basic Medicine and Cancer (IBMC), Chinese Academy of Science, 1# Banshan East Road, Hangzhou, 310022 Zhejiang China; 2 Department of Radiology, The Second Affiliated Hospital of Soochow University, 1055# Sanxiang Road, Suzhou, 215000 China; 3 Department of Radiology, The First Affiliated Hospital of SooChow University, 188#, Shizi Road, Suzhou, 215006 Jiangsu China; 4 Department of Radiology, Taizhou People’s Hospital of Jiangsu Province, 10# Yigchun Road, Taizhou, 225300 Jiangsu China; 5 Suzhou Institute of Biomedical Engineering and Technology, Chinese Academy of Sciences, 88# Keling Road, Suzhou, 215163 Jiangsu China

**Keywords:** Radiomics, Clinically significant prostate cancer, PI-RADS 3, Machine learning

## Abstract

**Purpose:**

To develop machine learning-based radiomics models derive from different MRI sequences for distinction between benign and malignant PI-RADS 3 lesions before intervention, and to cross-institution validate the generalization ability of the models.

**Methods:**

The pre-biopsy MRI datas of 463 patients classified as PI-RADS 3 lesions were collected from 4 medical institutions retrospectively. 2347 radiomics features were extracted from the VOI of T2WI, DWI and ADC images. The ANOVA feature ranking method and support vector machine classifier were used to construct 3 single-sequence models and 1 integrated model combined with the features of three sequences. All the models were established in the training set and independently verified in the internal test and external validation set. The AUC was used to compared the predictive performance of PSAD with each model. Hosmer–lemeshow test was used to evaluate the degree of fitting between prediction probability and pathological results. Non-inferiority test was used to check generalization performance of the integrated model.

**Results:**

The difference of PSAD between PCa and benign lesions was statistically significant (*P* = 0.006), with the mean AUC of 0.701 for predicting clinically significant prostate cancer (internal test AUC = 0.709 vs. external validation AUC = 0.692, *P* = 0.013) and 0.630 for predicting all cancer (internal test AUC = 0.637 vs. external validation AUC = 0.623, *P* = 0.036). T2WI-model with the mean AUC of 0.717 for predicting csPCa (internal test AUC = 0.738 vs. external validation AUC = 0.695, *P* = 0.264) and 0.634 for predicting all cancer (internal test AUC = 0.678 vs. external validation AUC = 0.589, *P* = 0.547). DWI-model with the mean AUC of 0.658 for predicting csPCa (internal test AUC = 0.635 vs. external validation AUC = 0.681, *P* = 0.086) and 0.655 for predicting all cancer (internal test AUC = 0.712 vs. external validation AUC = 0.598, *P* = 0.437). ADC-model with the mean AUC of 0.746 for predicting csPCa (internal test AUC = 0.767 vs. external validation AUC = 0.724, *P* = 0.269) and 0.645 for predicting all cancer (internal test AUC = 0.650 vs. external validation AUC = 0.640, *P* = 0.848). Integrated model with the mean AUC of 0.803 for predicting csPCa (internal test AUC = 0.804 vs. external validation AUC = 0.801, *P* = 0.019) and 0.778 for predicting all cancer (internal test AUC = 0.801 vs. external validation AUC = 0.754,* P* = 0.047).

**Conclusions:**

The radiomics model based on machine learning has the potential to be a non-invasive tool to distinguish cancerous, noncancerous and csPCa in PI-RADS 3 lesions, and has relatively high generalization ability between different date set.

## Introduction

Prostate cancer (PCa) is a global public health problem that threatens human health and life, which causes great harm to the male genitourinary system [1]. According to statistics from the American Cancer Research Association and the National Cancer Institute in 2019, PCa has become one of the most common malignant tumors in the world, accounting for the second most common malignancy in men [2]. Prostate Imaging Reporting and Data System (PI-RADS v2.1) published by American College of Radiology in 2019, represents a standardized method for assessing and reporting prostate MRI, which categorizes prostate lesions into different classes to reflect their relative likelihood of clinically significant prostate cancer (csPCa) [3]. PI-RADS 3 lesions included benign lesions and malignant lesions with different invasiveness and due to the absence of a clear tendency diagnosis for PI-RADS 3 lesions, there is a great variability in the practice patterns of different institutions (from conservative treatment, imaging follow-up to targeted biopsy), expense and potential clinical results [4]. Studies on evaluating the possibility of csPCa in targeted biopsies among PI-RADS 3 lesions have reported that cancer diagnosis rates range from 5 to 30%, and most studies have suggested that the likelihood of eventual diagnosis of csPCa is relatively low[5–7]. Therefore, accurately judging the benign and malignant lesions is helpful to reduce the pain caused by unnecessary biopsies.

Imaging monitoring without intervention for PI-RADS 3 lesions will undoubtedly reduce unnecessary biopsies. However, this method may lead to omission or delay in the diagnosis of csPCa lesions, resulting in irreversible consequences for patients. There is still controversy over whether to intervene in this “amphibolous lesions”[8], and the small but not insignificant proportion of lesions that represent csPCa, it is critical that a more detailed classification of the PI-RADS 3 lesions will benefit patients from biopsies and more aggressive treatment. Radiomics can convert images to higher-dimensional data, extract a large number of phenotypic features, and evaluate the biological behavior of tumor noninvasively through machine learning (ML) algorithms. It has been widely used in the diagnosis, invasiveness evaluation and clinical decision-making of PCa[9–11]. The number of radiomics studies focusing on PI-RADS 3 lesions is limited. Only two single-center studies have previously assessed the role of radiomics characteristics to detect cancer in these “equivocal lesions”. However, there are doubts about the universality and wide applicability of radiomics models in the absence of multi-institution trials. Therefore, the purpose of this work was to construct a ML-based radiomics model, which combined T2WI, DWI and ADC radiomics features, through a multi-center retrospective case–control study to validate its performance in differentiating PI-RADS 3 lesions from benign to malignant and in further risk stratification.

## Materials and methods

### Study design

This retrospective multi-agency study was approved by the ethics review committee of each participating institution and exempted from the need for informed consent of the patient. Four medical centers have signed data sharing agreements for data exchange (2021; Approval No. 262). All prostate MRI images from January 2018 to December 2019 were exported from each participating unit's PACS system. We summarized the data of each hospital, and there were a total of 2259 cases. 96 cases were excluded due to the absence of dynamic contrast enhanced MRI (DCE-MRI) and lack of pathological data, then the remaining 2163 cases were divided into two parts and graded according to PI-RADS v2.1 multiparametric MRI criteria [3] by two radiologists with 3 years of experience in prostate MRI diagnosis, who were blind to pathological findings when reading. While interpreting the images, two radiologists recorded the location of each lesion using the anatomical fan map recommend by PI-RADS v2.1 to correspond to the lesion described by the pathological results. At an interval of two weeks after the first assessment, the procedure was repeated by two readers and reviewed by a senior radiologist proficient in MRI diagnosis of the urinary system. When there was any dispute over the interpretion, the three discussed it until consensus was reached. Of the 2163 cases with final score results, 876 cases (40.5%) were classified as PI-RADS 1 and 2, 792 cases (36.6%) were classified as PI-RADS scores 4 and 5, and the remaining 495 cases (22.9%) were conferer with PI-RADS 3. Then, all PI-RADS 3 cases were selected for analysis, of which 32 were excluded based on the following criteria: (1) PI-RADS 3 lesions coexisted with other categories of lesions or doesn't match the targeted biopsy results; (2) prior to MRI examination, they had received intervention such as biopsy, surgery or hormone therapy; (3) lack of any clinical characteristics of the patient or poor image quality. Finally, 463 eligible patients were recruited and MRI images of each patient showed only one lesion.

**Fig. 1:**
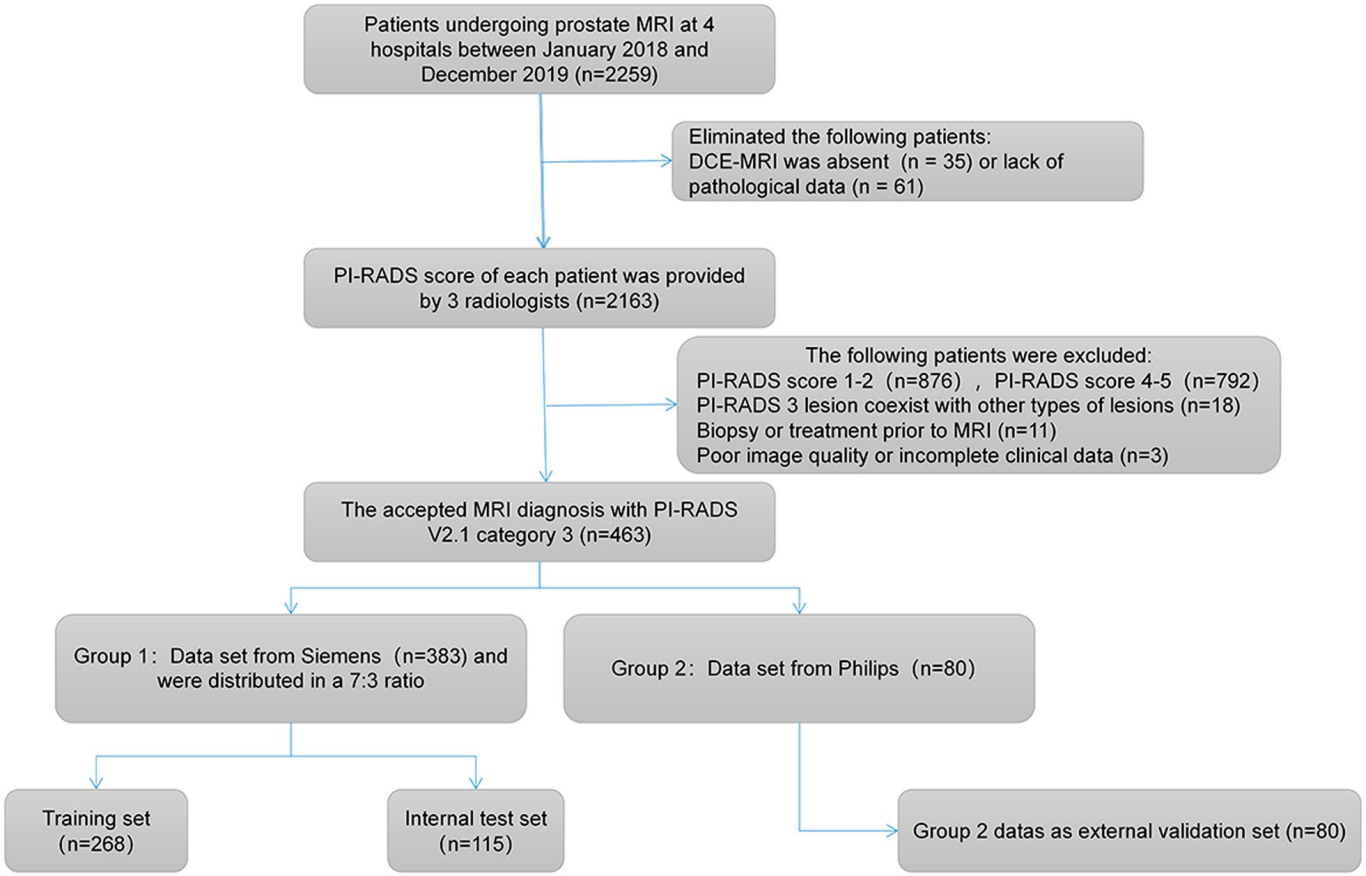
Flow diagram on methods of this study

All the screened cases were divided into two groups according to the supplier of scanning equipment. The first group included institutions 1–3 with a total of 383 patients, which were examined with 3.0 T superconducting MRI scanner (MAGNETOM Skyra, Germany) and equipped with 8-channel phased array body coils to collect signals. The second group consists of institution 4, with a total of 80 patients using a Dutch Philips Ingenia 3.0 T MRI scanner, the receiving coil was a 32-channel body phased array coil. The scanning sequences included T1WI, axial T2WI (no fat-saturated), sagittal T2WI, DWI (b = 100, 1000, 1500, 2000 s/mm^2^) and DCE-MRI. The ADC value was calculated by a single exponential signal attenuation model based on the DWI images with b = 100 and 1000 s/mm^2^. During DCE scanning, 15 to 20 slices were scanned once, the scanning time resolution was 5.8 s, 64 phases were scanned, and the scanning time was 7 min. After the end of the third dynamic scanning phase, contrast agent gadolinium meglumine pentanoate was injected intravenously at the injection rate of 3 ml/s and the dose of 0.1 mmol/kg. MRI scan parameters are described in Table 1. The cases of the first group were randomly divided into training set (n = 268) and internal test set (n = 115) according to the proportion of 7:3. The second group of cases was used as an external validation set (n = 80) to evaluate the extensibility of the model. Figure 1 provides a flowchart that includes patient selection and case assignment.

**Table 1:** MRI protocols for both vender

MRI vendor sequence	Siemens Skyra 3.0 T MR scanner (Germany)				Philips Ingenia 3.0 T MR scanner (Netherlands)			
	T1WI	AxialT2WI	SagittlT2WI	DWI	T1WI	AxialT2WI	SagittlT2WI	DWI
TR(ms)	680.0	6980.0	3900.0	5000.0	556.0	3000.0	4978.0	6000.0
TE(ms)	13.00	104.00	89.00	72.00	8.00	100.00	100.00	77.00
Slice thickness(mm)	5.0	3.0	3.0	3.0	5.0	3.0	1.5	3.0
Slice gap(mm)	0.50	0.00	0.45	0.00	0.00	0.00	0.15	0.00
Matrix	384 × 384	384 × 384	384 × 384	130 × 130	276 × 406	240 × 161	276 × 238	124 × 121
FOV(mm × mm)	380 × 380	200 × 200	200 × 200	288 × 288	249 × 415	220 × 220	240 × 180	220 × 220
NSA	1	2	3	2	1	3	2	2

TR repetition time; TE echo time; NSA number of signal averaged; T1WI T1 weighted imaging; T2WI T2 weighted imaging; DWI Diffusion Weighted Imaging

### Targeted biopsy and histopathology

MRI-TRUS fusion targeted sample was performed with Hitachi real-time ultrasonic multi-image fusion navigation system (RVS), and the machine model was HIVISIONNoblus/TopicPath. The suspicious lesions were sampled by MRI-TRUS fusion biopsy and systematic biopsy under the guidance of TRUS within 4 weeks after the MRI examination. Before the fusion biopsy, the original data of prostate MRI in DICOM format were introduced into the main body of RVS ultrasound. MRI images were fused with TRUS images after general anesthesia, and anatomical markers such as urethra orifica, urethra, mullerian or ejaculatory duct cyst were matched with MRI sagittal images on the same section. T2WI, DWI, or DCE images with significant abnormal signals were selected to mark the target lesions in the cross-sectional MRI, while the same ultrasound sites were labeled (convex array scan), and then switched to sagittal images to further confirm the synchronization of MRI and ultrasound. After confirming favourable synchronization of MRI-TRUS images, the sagittal plane of prostate was taken by TRUS, and the target lesion marked with “ + ” was found. Under the guidance of puncture stent, the 18G disposable puncture gun was used to insert needle through perineum and the puncture gun was fired close to the target center. Then, the axial plane scan was converted to confirm that the needle track enters the target. 2–4 needles were punctured for each suspicious focus. After the targeted sample, 12-needle systematic biopsy was conducted through perineum under the guidance of TRUS, and all the pathological specimens were marked in detail according to each partition and fixed with 10% formaldehyde for pathological examination.

The pathological results were evaluated by urological pathologist independently of the MRI results, and the location and boundary of the lesions were recorded to ensure that they correspond to the suspicious lesions on MRI images. The grade grouping and Gleason score of the lesions were determined according to the 2014 ISUP guidelines. csPCa was defined as ISUP grade 2 or higher (Gleason = 3 + 4 or higher), and pathological results with GS = 3 + 3 (ISUP grade 1) were defined as clinically insignificant PCa (ciPCa) [12].

### Focus segmentation

Subsequent radiomics analysis were performed based on axial T2WI, DWI (b = 2000 s/mm^2^) and ADC images in our study. The different target images of the same patient were spatially matched using Elastix software package (v.4.10, 13. Using T2WI images as reference, DWI and ADC images were registered successively. Lesion segmentation was performed jointly by two radiologist involved in imaging evaluation using ITK-SNAP 3.8.0 software (http://www.itksnap.org/). The two handlers drew the region of interest (ROI) layer by layer on T2WI sequence to get the volume of interest (VOI) of the tumor, then copy it to DWI and ADC images to ensure the consistency of VOI sketches in different sequences. After preprocessing, visually verified was performed by a professor with experience in prostate MRI diagnosis (more than 10 years) to ensure that the location and extent of the lesions shown on MRI strictly matched the corresponding pathological description.

### MRI image preprocessing and feature extraction

Before the feature calculation, the images of each patient were standardized separately to improve the texture recognition rate. Firstly, the T2WI, DWI and ADC images of each patient were resampled to a voxel size of 1 × 1 × 1 cm^3^ to standardize the voxel spacing. Then the voxel intensity discretization was accomplished by setting the bin width to 25 to reduce imaging noise and standardize the intensity. Finally, through Z-score Normalization for different sequence of each case, which can reduce the influence of the inconsistency of image parameters on the variation of radiomics features, the voxel intensity was transformed into a distribution with 0 as the mean and 1 as the standard deviation.

The open source radiomics software FeAture Explorer (FAE v0.4.0), which based on pyradiomics package, was used to extract features from the VOI of each sequence [14]. According to the 8 texture analysis methods provided by the software, a total of 2347 radiomics features were extracted from ROI files: (1) 46 first-order gray statistics; (2) 38 shape-based features; (3) 70 Gray Level Co-occurrence Matrices (GLCM); (4) 20 Gray Level Run Length Matrices (GLRLM); (5) 42 Gray Level Size Zone Matrices (GLSZM); (6) 36 Gray Level Dependence Matrices (GLDM); and (7) 17 Neighborhood Gray Tone Difference Matrices (NGTDM). (8) The original images were transformed by Wavelet Transform, and 2078 wavelet features are extracted in three spatial directions. The repeatability of intra- and inter-observer of lesion segmentation was based on the repeatability of feature extraction. 30 patients were randomly selected and the clinical data were blinded. The two doctors performed VOI segmentation and feature extraction again. The intra- and inter-observer repeatability of feature extraction was evaluated by intergroup correlation coefficient (ICC). If the intra-group and inter-group correlation coefficient is greater than 0.75, it is considered that the ROI drawing has acceptable stability.

### Feature selection and classifier modeling

In this study, we focus on two results: (1) distinguish any cancer lesions from benign lesions, (2) and further predict csPCa occurrence in all cases. In order to solve the problem of sample imbalance in the training set, this study used the synthetic minority oversampling technique (SMOTE) to oversample the positive samples to balance with negative samples. Internal test set and external validation set did not perform this process [15]. The number of radiomics features was much larger than the number of samples, which may increase the risk of overfitting. This risk was reduced by feature selection to reduce the number of features. In present study, Z-score Normalization was first used to normalize the feature matrix, each feature vector was subtracted from the mean value and divided by the standard deviation to eliminate the order of magnitude otherness between different features. The radiomics features with variance of 0 were eliminated, and then the data dimension was reduced to remove the redundant features with average Spearman absolute correlation coefficient ≥ 0.9. After eliminated redundant features, the analysis of variance (ANOVA) algorithm was used to sort the features, and only the top 20 features were retained. These features with increments from 1 to 20 were then input into the support vector machine (SVM) classifier. For different sequences, T2WI, DWI and ADC feature matrices were modeled respectively (called T2WI-model, DWI-model and ADC-model), and then the features of the three sequences were combined for modeling analysis (call integrated model). While established models to identify csPCa, the features of the first group of cases were re-integrated, mean that, the benign lesions and ciPCa were divided into the same label with their features. Then the reconstituted cases was divided into training set (n = 268) and internal test set (n = 115) according to the proportion of 7:3, and the generalization ability of the model was verified on the external valitation set. All the experiments above were run in FeAtureExplorer.

### Statistical analysis

Demographic datas were compared by chi-square test and independent t-test. According to whether it conformed to the normal distribution, the quantitative data were expressed as average (± standard deviation) or median (quartile range), *P* < 0.05 was considered statistically significant. Prediction models were inspected on the internal test and external validation sets. The receiver operating characteristic (ROC) curve was analyzed and the area under the ROC curve (AUC) was quantified to evaluate their performance in distinguishing cancer from benign lesions. Hosmer–lemeshow test was used to evaluate the degree of fitting between the predicted results of the integrated model and the histopathological results, and drawn the calibration diagram to visually display the results. In order to evaluate the generalization ability of the model, the non-inferior test was used to check whether the AUC of the external validation set is not lower than that of in the internal test set. R software (version 4.1.0, www. Rproject. org) was uesd for non-inferiority testing, the predefined acceptable threshold value was set to 0.1. Through the non-inferiority test of each model, the P-value was obtained, when *P* < 0.05, it indicates that the model has good versatility.

## Results

Clinical characteristics included age, prostate specific antigen (PSA), prostate volume (PV) and PSA-density (PSAD). The mean age, PSA, PV and PSAD of patients were 62.6 ± 8.2 years, 8.92 (6.78–14.26) ng/mL, 35.23 (27.24–42.59) mL and 0.22 (0.17–0.84) ng/mL^2^, respectively. Of the 463 PI-RADS 3 lesions, 311 (67.2%) were benign and 152 (32.8%) were PCa lesions, of which 11.2% (52/463) were ciPCa (ISUP grade 1), 21.6% (100/463) were csPCa (47 ISUP grade 2, 20 ISUP grade 3, 23 ISUP grade 4, 10 ISUP grade 5). PSAD in benign lesion group and PCa group were 0.17 (0.09–0.41) and 0.39 (0.16–1.08), respectively (*P* = 0.006). There was no difference in the distribution of PCa and csPCa between different institutions (*P* = 0.502, 0.173). From the 463 PI-RADS 3 lesions, there were 216 peripheral zone lesions (46.7%) with 79 PCa (48 csPCa and 31 ciPCa) and 247 transition zone lesions (53.3%) with 73 PCa (52 csPCa and 21ciPCa). The patient's demographic and clinical datas were shown in Table 2.

**Table 2:** The patient’s demographic and clinical datas among benign lesion and prostate cancer

	Benign lesion	Prostate cancer	P-value
Number of cases, n (%)	311 (67.2%)	152 (32.8%)	
Ages (years), mean [SD]	61.5 ± 11.7	66.7 ± 5.9	0.079^a^
PSA (ng/mL), median (IQR)	8.37 (6.45–11.07)	9.12 (7.05 ~ 18.34)	0.083^a^
PV (mL), median (IQR)	37.65 (28.43 ~ 44.29)	34.72 (26.35 ~ 41.28)	0.425^a^
PSAD (ng/mL^2^), median (IQR)	0.17 (0.09 ~ 0.41)	0.39 (0.16 ~ 1.08)	0.006^a^
*Biopsy results, n*			
ISUP grade 1 (ciPCa)		52	
ISUP grade 2		47	
ISUP grade 3		20	
ISUP grade 4		23	
ISUP grade 5		10	
Lesion location, n (%)			0.747^b^
Peripheral zone Transition zone	137 (29.6%) 174 (37.6%)	79 (17.1%) 73 (15.7%)	

*PSA* prostate specific antigen; *PV* prostate volume; *PSAD* PSA-density; *ciPCa* clinically insignificant prostate cancer

^a^Independent t-test; ^b^Chi-square test

**Fig. 2:**
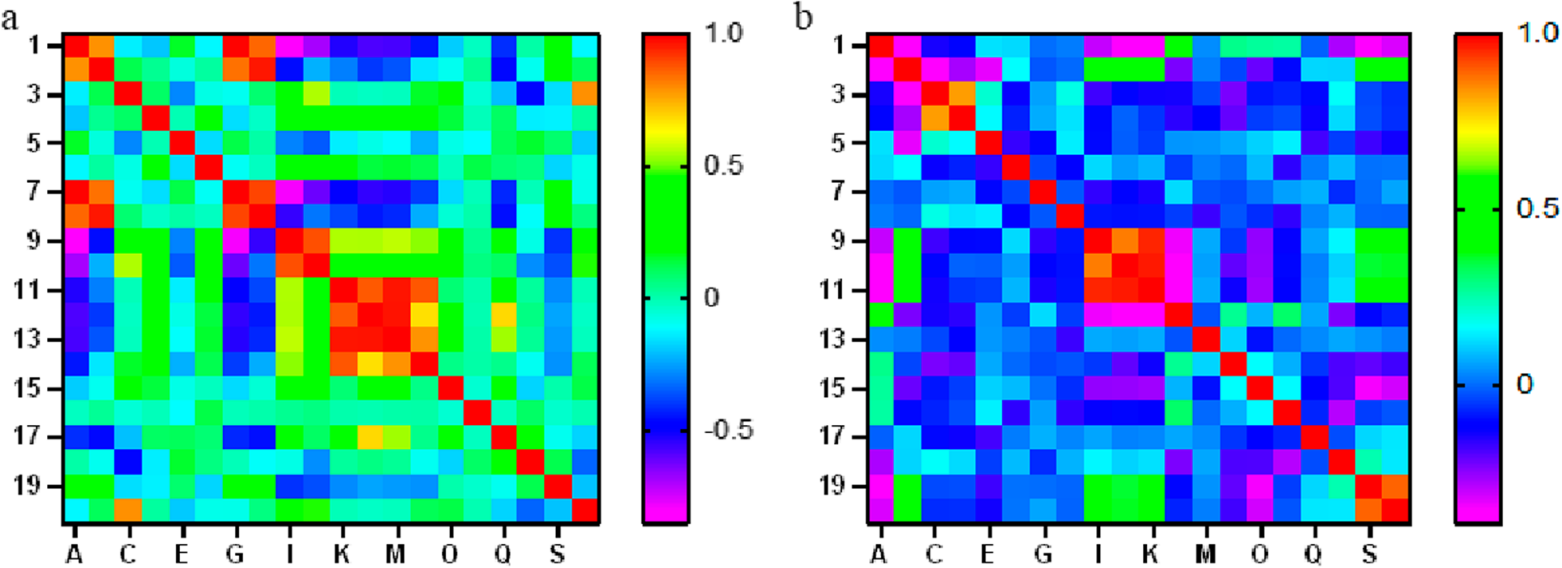
Determine the number of features used to construct the prediction model for differential diagnosis between benign and malignant lesions (**a**) and further identification csPCa in cancer lesions (**b**)

**Fig. 3:**
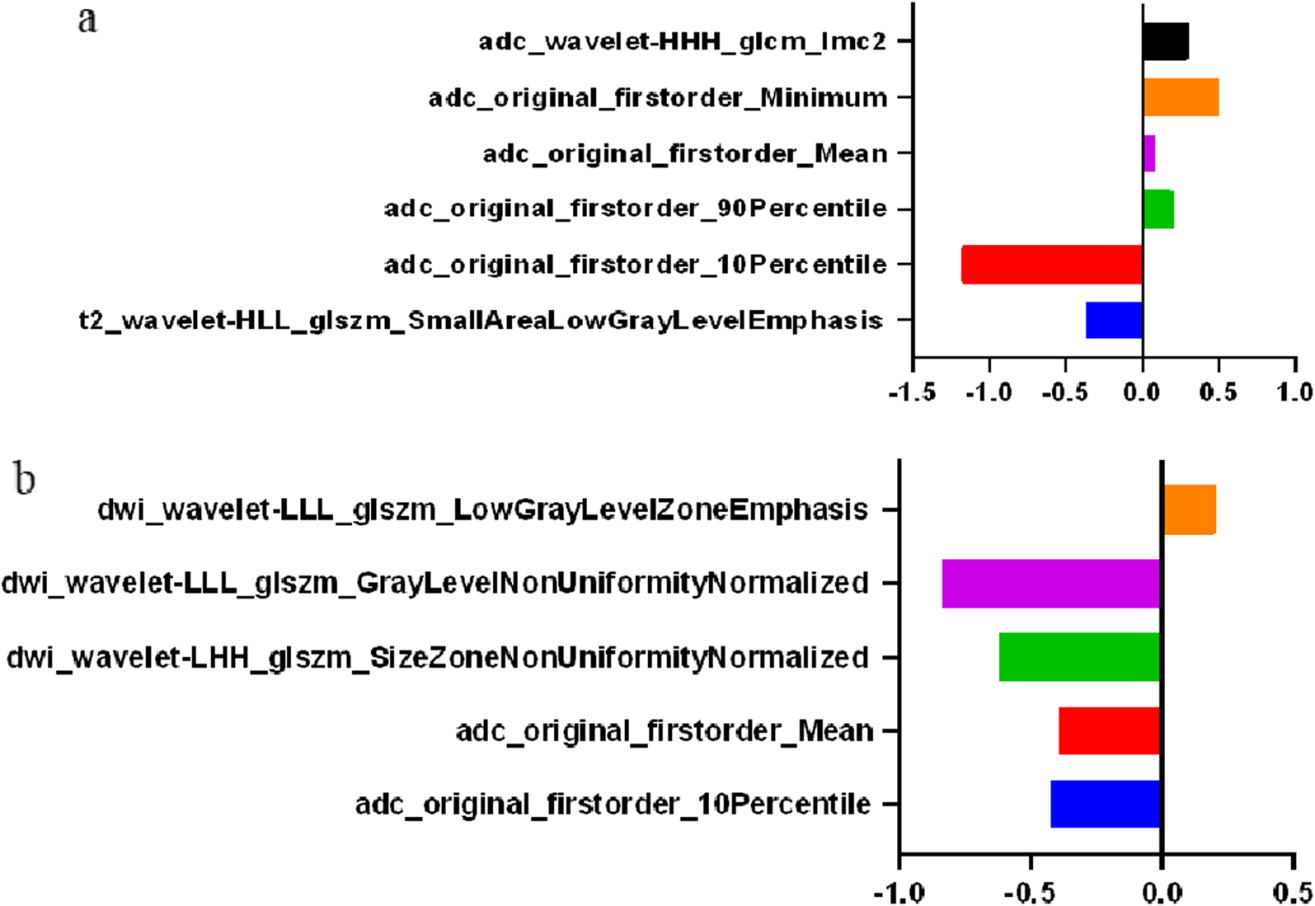
Feature names and coefficients in models for differential diagnosis between benign and malignant lesions (**a**) and further identification csPCa in all lesions (**b**)

In the intra- and inter-observer consistency test, the intra-observer ICCs range was 0.77–0.90, and the inter-observer ICCs range was 0.80–0.87, indicated that the repeatability of feature extraction was fine. Spearman correlation test results of the top 20 features screened by ANOVA were represented by feature heatmap (Fig. 2). While constructed the integrated model, 6 and 5 features were screened to distinguish benign from malignant lesions and to further identify csPCa in all lesions. The name of the features and the corresponding coefficient are shown in Fig. 3.

The accuracy of PSAD in identifying csPCa of PI-RADS 3 lesions was 0.652 and 0.650 in internal test and external validation set, respectively, and the mean AUC value was 0.701 (internal test AUC = 0.709, external valitation AUC = 0.692, *P* = 0.013). The accuracy of the model in distinguishing benign and malignant PI-RADS 3 lesions in internal test and external validation set was 0.583 and 0.575, respectively, with mean AUC of 0.630.

The accuracy of T2WI-model in identifying csPCa of PI-RADS 3 lesions was 0.774 and 0.763 in internal test and external validation set, respectively, and the mean AUC value was 0.717 (internal test AUC = 0.738, external valitation AUC = 0.695, *P* = 0.264). The accuracy of the model in distinguishing benign and malignant PI-RADS 3 lesions in internal test and external validation set was 0.643 and 0.650, respectively, with mean AUC of 0.634.

The accuracy of DWI-model in identifying csPCa of PI-RADS 3 lesions was 0.730 and 0.813 in internal test and external validation set, respectively, and the mean AUC value was 0.658 (internal test AUC = 0.635, external validation AUC = 0.681, *P* = 0.086). The accuracy of the model in distinguishing benign and malignant PI-RADS 3 lesions in internal test and external validation set was 0.730 and 0.638, respectively, with mean AUC of 0.655.

The accuracy of ADC-model in identifying csPCa of PI-RADS 3 lesions was 0.739 and 0.775 in internal test and external validation set, respectively, and the mean AUC value was 0.746 (internal test AUC = 0.767, external validation AUC = 0.724, *P* = 0.269). The accuracy of the model in distinguishing benign and malignant PI-RADS 3 lesions in internal test and external validation set was 0.565 and 0.613, respectively, with mean AUC value of 0.645.

The integrated model based on three single-sequence radiomics features, and its accuracy in identifying csPCa was 0.748 in internal test set and 0.863 in external validation set. The mean AUC value was 0.803 (internal test AUC = 0.804, external validation AUC = 0.801, *P* = 0.019). The accuracy of the model in distinguishing benign and malignant PI-RADS 3 lesions in internal test and external validation set was 0.748 and 0.763, respectively, with mean AUC of 0.778. The results of Hosmer–Lemeshow test showed that the prediction results of the integrated model for all PCa and csPCa in the internal test and the external validation set had a high coincidence rate with the observed risks (*P* = 0.073 vs. 0.082 for PCa; *P* = 0.224 vs. 0.647 for csPCa, respectively).

**Fig. 4:**
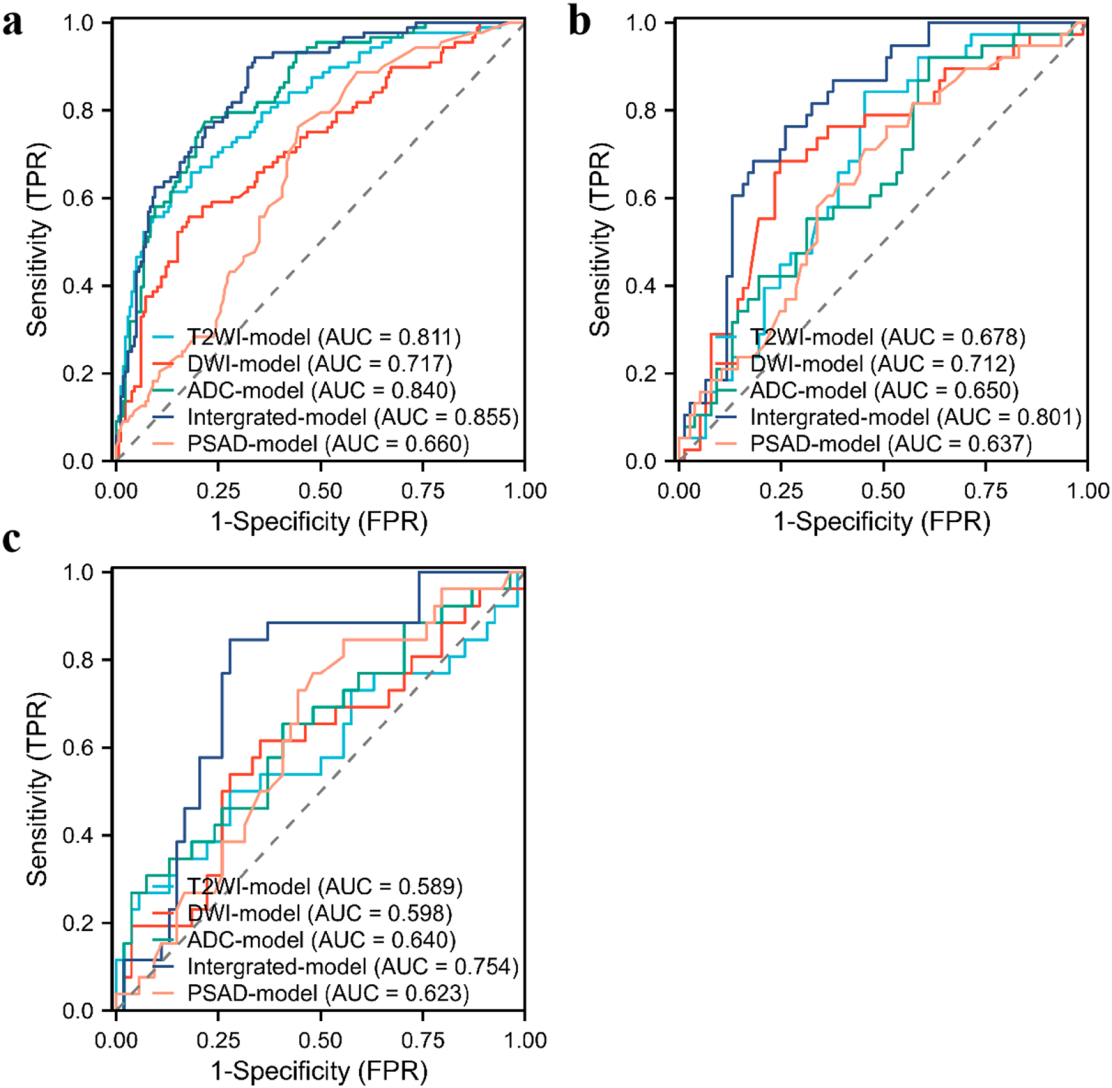
The ROC curves of PSAD and four models in predicting any tumor in PI-RADS 3 lesions. **a** training set, **b** internal test set, **c** external validation set

**Fig. 5:**
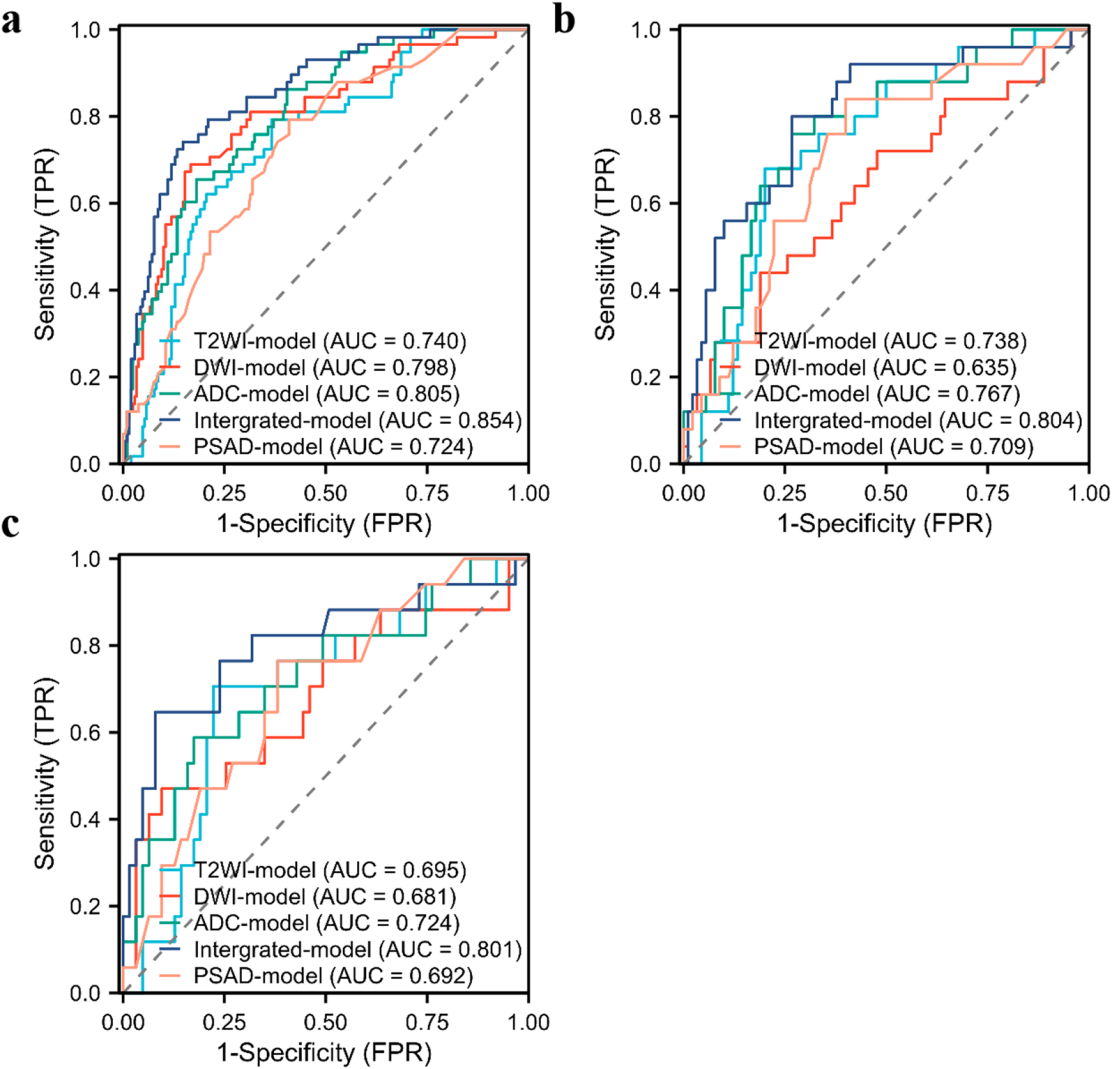
The ROC curves of PSAD and four models in predicting csPCa in PI-RADS 3 lesions. **a** training set, **b** internal test set, **c** external validation set

**Fig. 6:**
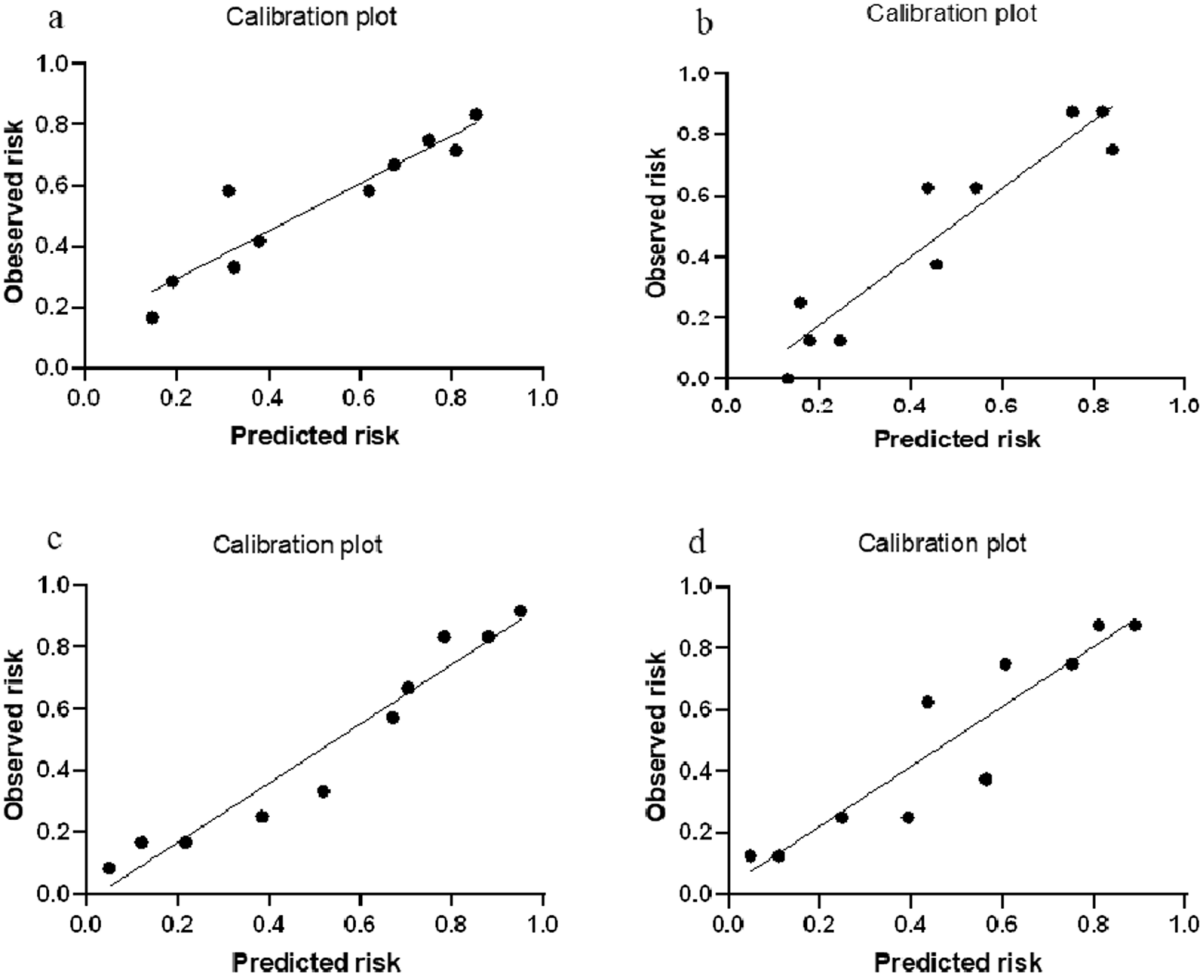
The calibration plots of joint model in predicting all PCa (**a**, **b**) and csPCa **c**, **d** in PI-RADS 3 lesions. (a, c)internal test set, **b**, **d** external validation set

The results of each model for distinguishing benign and malignant PI-RADS 3 lesions are shown in Table 3, and the corresponding ROC curves are shown in Fig. 4. The effectiveness of each model in identifying csPCa are compared in Table 4, and the corresponding ROC curves are shown in Fig. 5. The pathological calibration scatter plots of the prediction results of the integrated model are shown in Fig. 6.

**Table 3:** The performance of each model for predicting any tumors in PI-RADS 3 lesions

Modality	Training set				Internal test set				External validation set				Mean AUC*	*P* value
	AUC	ACC	SEN	SPE	AUC	ACC	SEN	SPE	AUC	ACC	SEN	SPE		
T2WI-model	0.811	0.784	0.614	0.867	0.678	0.643	0.842	0.545	0.589	0.650	0.500	0.722	0.634	0.547
DWI-model	0.717	0.735	0.557	0.822	0.712	0.730	0.684	0.753	0.598	0.638	0.616	0.648	0.655	0.437
ADC-model	0.840	0.780	0.773	0.783	0.650	0.565	0.921	0.390	0.640	0.613	0.654	0.593	0.645	0.848
Integrated-model	0.855	0.746	0.920	0.661	0.801	0.748	0.763	0.740	0.754	0.763	0.846	0.722	0.778	0.047
PSAD-model	0.660	0.623	0.761	0.556	0.637	0.583	0.763	0.494	0.623	0.575	0.846	0.444	0.630	0.036

*T2WI* T2 weighted imaging; *DWI* diffusion weighted imaging; *ADC* apparent diffusion coefficient; *PSAD* PSA-density; *AUC* area under the receiver operating characteristic curve; *ACC* accuracy; *SEN* sensitivity; *SPE* specificity

^*^Mean AUC = [AUC(Internal test set) + AUC(External validation set)]/2

The *P* values from the non-inferiority tests

**Table 4:** The performance of each model for predicting csPCa in all PI-RADS 3 lesions

modality	Training set				Inner test set				External validation set				Mean AUC*	*P* value
	AUC	ACC	SEN	SPE	AUC	ACC	SEN	SPE	AUC	ACC	SEN	SPE		
T2WI-model	0.740	0.668	0.793	0.633	0.738	0.774	0.680	0.800	0.695	0.763	0.706	0.778	0.717	0.264
DWI-model	0.798	0.802	0.690	0.833	0.635	0.730	0.440	0.811	0.681	0.813	0.471	0.905	0.658	0.086
ADC-model	0.805	0.784	0.655	0.819	0.767	0.739	0.760	0.733	0.724	0.775	0.588	0.825	0.746	0.269
Integrated-model	0.854	0.828	0.741	0.852	0.804	0.748	0.800	0.733	0.801	0.863	0.647	0.921	0.803	0.019
PSAD-model	0.724	0.634	0.793	0.590	0.709	0.652	0.840	0.600	0.692	0.650	0.765	0.619	0.701	0.013

*csPCa* clinically significant prostate cancer; *T2WI* T1 weighted imaging; *DWI* diffusion weighted imaging; *ADC* apparent diffusion coefficient; *PSAD* PSA-density; *AUC* area under the receiver operating characteristic curve; *ACC* accuracy; *SEN* sensitivity; SPE specificity

*Mean AUC = [AUC(Internal test set) + AUC(External validation set)]/2

The P-values from the non-inferiority tests

## Discussion

This study shows that radiomics models based on ML algorithm, which used T2WI, DWI and ADC radiomics features, can achieve upper-moderate accuracy when predicting any cancer and csPCa in PI-RADS v2.1 3 lesions, and the performance of integrated model is better than that of all single-sequence models, which indicates that only based on the simplex radiomics feature may be limited in distinguishing significant tumors from benign or inert lesions, and the combination of multiple features is well complementary. However, it is worth noting that the performance of all models in predicting csPCa is better than that of models in predicting all cancers. Therefore, our results also show that the heterogeneity of csPCa is more obvious than that of ciPCa, and it is easier to be recognized in ML progress.

Several additional indicators have been introduced to predict the need for biopsy in patients with PI-RADS 3, including lesion size, PV, ADC, PSA and PSAD, but the published results do not fully prove the relationship between these indicators and the risk of csPCa appearance [16–19]. For example, quantitative ADC values can help detect carcinoma while avoiding biopsies that are negative [20]. However, another study showed that the difference of median ADC values in PI-RADS 3 lesions was not statistically significant [18]. Zhang et al. [21] showed that age, PSAD, lesion zone and ADC value were Independent predictors for differentiating csPCa and non-csPCa. Used PSAD as a benchmark, this study compared the diagnostic efficacy of radiomics and clinical indicators, and the results showed that the mean AUC of integrated model was higher than that of PSAD (0.803 vs. 0.701), and accuracy was greatly improved, indicating the superiority of radiomics as a valuable alternative to more simple and already recognized quantitative parameters.

In recent years, radiomics studies have mainly focused on tumor detection, prediction of PI-RADS score and Gleason grade, evaluation of tumor extra-capsular extension and therapeutic response, which have shown similar performance as PI-RADS [22, 23]. However, there are few studies use radiomics to assisted diagnose PI-RADS 3 lesions, and lack multi-center studies to validate the generalization ability of the model. Our results show that the single-sequence model is less efficient in both internal test and external validation set, with the lowest mean AUC for T2WI radiomics features, which is similar to the results of Lim et al. [24]. They constructed a model based on XGBoost algorithm to predict any cancer or csPCa in PI-RADS 3 lesions, and AUC performed by T2WI features for all types of tumor was 0.608 and 0.547 for csPCa, lower than 0.642 and 0.684 of ADC features. Hectors et al. reconfirmed that model with T2WI radiomics features had a low ability to diagnose csPCa (AUC = 0.76) [25]. However, the radiomics features of ADC and DWI images were not included as controls in their study. Our results are lower than those of Hou et al. [26], who extracted features from T2WI, DWI and ADC images, constructed a one-step ML model and a regression analysis model integrated radiomics score, and improved the risk stratification method for identifying csPCa in PI-RADS 3 lesions with AUC reached 0.74–0.89.

There are several design differences between this study and previous studies, which may explain the conflicts in results with Hou and Hectors. In contrast to these studies, our study used MRI datas from two vendors in four medical units. Different MRI scanners are equipped with different software and hardware, and these differences mean that scanners may not obtain images with the same intensity distribution [27–29]. This is why we performed resampling, gray discretization and Z-score normalization prior to radiomics feature extraction. In order to demonstrate that image standardization can reduce the distraction of multi-center datas on the performance and generalization ability of machine learning model, an independent external validation set was set up to evaluate the model's performance, which cases was provided by a different supplier from the testing set. The models constructed by Hou et al. and Hectors et al. were trained and tested only in their respective institutions, which limited extensibility. For example, quantitative values of DWI and ADC may be affected by variabilities between different scanners, imaging parameters, and patients, which caused the repeatability controversial. The lower accuracy of our study may be due to the fact that datas from multiple centers were integrated together and the number of PCa contributed by each participating unit was different, leading to differences in the distribution of cases. In order to ensure consistency between the combined data set and the distribution of cases in a single center, Lim et al. conducted a subgroup analysis of larger disease-causing institutions, but was unable to confirm this conjecture. Our study used non-inferiority test to evaluate the model's generalization ability, which was not available in other studies. Although we failed to prove that the AUC of all single-sequence radiomics featuers in the external validation set was not lower than that in the internal test set (*P* > 0.05). However, the diagnostic accuracy and sensitivity of the integrated model in external validation set are higher than that of the internal test set, and the AUC in external validation set was not inferior to the AUC in internal test set (*P* < 0.05), indicating that the integrated model has certain generalization ability in different date sets. In addition, Ji et al. [30] constructed a comprehensive model combinie age, PSA and radiomics features, suggested that combin clinical features can improve the generalization ability of radiomics model. Different reference standards may also be one of the reasons for the different results. In Hou et al. 's study, a subset of included lesions lacked pathological diagnosis, and the clinical significance of tumor foci was only inferred based on follow-up imaging results and/or PSA changes after empirical treatment. This limits the reliability of the model's results for predicting a subset of clinically significant cancers, some of which were misclassified when they could have been monitored closely [31].

For the single-sequence model, radiomics features extracted from DWI/ADC sequence have better performance than T2WI features in distinguishing between benign and malignant lesions. This is consistent with the research of Hou et al. In anther similar study, the most important feature for detecting tumor in PI-RADS 3 lesions was based on ADC images [32]. The changes of diffusion of water molecules in tissues were monitored by DWI images, reflecting the changes of cell volume and number in epithelium, stroma and luminal space [33]. PCa is high cellular tissue, which restricts the diffusion to some extent due to the blocking of the random movement of water molecules in the tumor. The degree of diffusion limitation is positively correlated with the tumor grade, invasiveness and stage [34]. ML can quantify subtle changes in the diffusion motion of water molecules in the DWI/ADC diagram, which makes diffusion imaging perform better than other sequences to evaluate the PCa.

There are several limitations in this study. First, this is a retrospective case–control study with a relatively small sample size, especially for a small number of csPCa with uneven distribution between groups, may be at risk of over-fitting when training models, which limits the evaluation of the accuracy in predicting malignant tumor. Second, The imaging-pathology correlation was based on targeted biopsy pathology. However, part of the biopsies occurred before the release of PI-RADS v2.1, resulting in differences between the lesions indicated by targeted biopsies and the PI-RADS 3 lesions after reassessment. A retrospective correlation with pathology could only be possibly with radical prostatectomy specimens. Third, the location of the lesion, such as peripheral and transitional zones, or poorly defined areas, was not taken into account. Due to the differences between peripheral zones and transition zones, modeling for each region may affect model performance. Finally, it was not discussed whether the clinical factors combined with radiomics features can provide additional diagnostic value for PI-RADS 3 lesions.

## Conclusion

The ML-based radiomics model achieved an encouraging performance in differentiating PI-RADS 3 lesions from benign to malignant and distinguishing significant or indolent tumors, which has certain application value to assist clinical decision making, and provides a new direction for the management of patients with controversial MRI diagnosis and helps to reduce unnecessary biopsies while improving the detection rate of csPCa.

## Fundings

This research was supported by the Special Program for Diagnosis and Treatment Technology of Clinical Key Diseases in Suzhou (LCZX202001), Medical and Health Science and Technology Innovation Program in Suzhou (SKY2022003), Jiangsu Provincial Key Medical Discipline (JSDW202242).

## Data Availability

The datasets generated during this study are available from the corresponding author upon reasonable request.

## Abbreviations

PCa: Prostate cancer
PI-RADS: Prostate imaging reporting and data system
csPCa: Clinically significant prostate cancer
ciPCa: Clinically insignificant PCa
PSA: Prostate specific antigen
PV: Prostate volume
PSAD: PSA-density
ML: Machine learning
ROI: Region of interest
VOI: Volume of interest
ROC: Receiver operating characteristic
AUC: Area under curve
